# Federated learning and Data Lakehouse for healthcare analytics: a knowledge transfer initiative between Germany and Tunisia

**DOI:** 10.3389/fmed.2026.1759016

**Published:** 2026-04-21

**Authors:** Mohamed Ali Hadj Taieb, Mohamed Merdassi, Aymen Tlili, Mohamed Ali Bouri, Moheddine Ben Abdallah, Débora Gnuito, Houcemeddine Turki, Mohamed Habib Kammoun, Zied Ouerda, Mehrshad Jaberansary, Bruce Schultz, Fu-Sung Kim-Benjamin Tang, Adamantios Koumpis, Mohamed Ben Aouicha, Oya Beyan

**Affiliations:** 1 Data Engineering and Semantics Research Unit, Faculty of Sciences of Sfax, University of Sfax, Sfax, Tunisia; 2 Institute for Biomedical Informatics, University of Cologne, Medical Faculty and University Hospital Cologne, Cologne, Germany

**Keywords:** Data Lakehouse, federated learning, Germany, healthcare, knowledge transfer, Tunisia

## Abstract

Healthcare institutions worldwide generate growing volumes of heterogeneous clinical data, yet legal, ethical, and infrastructural constraints often prevent these data from being centralized for analysis. Federated learning approaches offer a promising solution by enabling multi-site computation without transferring sensitive patient information, but require well-designed cross-site data harmonization. Modern Data Lakehouse architectures address this requirement by providing a scalable, governed foundation for multimodal clinical dataset integration through unified storage, metadata-rich governance, and FAIR-aligned data access. Despite increasing interest in such technologies across the Middle East and North Africa (MENA) region, operational deployments remain limited due to fragmented infrastructures, insufficient data governance, and gaps in practical expertise. This perspective article reports on a German–Tunisian knowledge and technology transfer initiative conducted within the DAAD Ta’ziz Partnership programme. As mentioned in the, ‘the Arabic word ‘Ta’ziz’ means ‘strengthening/consolidation’ and has been chosen to clearly express the intended outcome of the programme’ [https://www.daad.de/en/information-services-for-higher-education-institutions/further-information-on-daad-programmes/taziz-partnership/ (visited on November 28th, 2025)]. The collaboration between the University Hospital of Cologne and the University of Sfax introduced and implemented federated learning concepts via the Personal Health Train paradigm, and explored the design of a Data Lakehouse tailored to emerging healthcare ecosystems in Tunisia. Through an internship programme, hands-on MLOps training, and a large-scale workshop, the project built technical capacity in containerized analytics workflows, data governance, FAIR data management, and lakehouse engineering. We synthesize lessons learned regarding infrastructural limitations, data governance maturity, interoperability challenges, and institutional readiness, and outline considerations for sustainable adoption of distributed analytics in the MENA region. The findings highlight the critical importance of capacity building, bidirectional knowledge exchange, proof-of-concept validation, and administrative engagement for deploying trustworthy AI and modern data infrastructures in sensitive healthcare environments. We by emphasizing the need for further developments regarding federated learning and Data Lakehouse adoption in Tunisia, and how cross-regional partnerships can accelerate responsible, privacy-preserving digital health innovation.

## 1 Introduction

Healthcare institutions across the world generate increasingly large and heterogeneous patient datasets through clinical tasks, ranging from general electronic health records to specific imaging data or laboratory measurements. Leveraging the generated data is vital for biomedical analytics, such as clinical decision support, and requires secure, scalable and interoperable infrastructures. Traditional centralized data architectures aggregate data from different healthcare institutions at a central location, which poses major legal, ethical and technical challenges. Data Privacy regulations, such as the European General Data Protection Regulation (GDPR) impose strict requirements for accessing or sharing sensitive health information, hindering cross-institutional data transfer and underscoring the need for novel privacy-preserving analytics approaches.

Distributed analytics, such as federated learning, emerged as a solution to enable analytics and machine learning model training across institutions without sharing sensitive patient data. Instead of transferring data to a central location for model training, analytical algorithms are transferred to each institution for local execution and only the aggregate results or model updates are returned. This paradigm, also known as the Personal Health Train (PHT) in healthcare (1) is implemented through various platforms such as Vantage6 (2), PHT-meDIC (3) or PADME (4) and facilitates the transfer of algorithms through the concept of trains to different institutions conceptualized as stations.

Leveraging data from different institutions, however, can lead to challenges regarding data harmonization, especially with the integration of multimodal data (5).

The integration of multimodal healthcare data can be facilitated through Data Lakehouse architectures, which transform how complex datasets can be stored, queried and analyzed through a standardized access point to combine the flexibility of data lakes storing multimodal data with the structure and governance of warehouses (6).

However, despite these recent advances, challenges regarding limited infrastructure, insufficient technical experience, fragmented healthcare IT ecosystems, and a lack of harmonized standards still exist. While interest in AI-driven health analytics is growing in the MENA region, operational deployments remain rare, as academic institutions often have strong computer science and engineering talent, yet limited exposure to real-world medical data workflows and regulatory frameworks.

To address this gap, the German Academic Exchange Service (Deutscher Akademischer Austauschdienst—DAAD) is awarding funds provided by the Federal Foreign Office to support the Ta’ziz Partnership programme with partner universities in the following countries in the MENA region (Middle East & North Africa): primarily Tunisia, Sudan, Lebanon and Iraq, as well as Egypt, Algeria, Yemen, Jordan, Libya and Morocco.^1^

Within this context, our team from the University Hospital of Cologne’s Biomedical Informatics Institute in Germany and the Data Engineering and the Semantics Research Unit at the University of Sfax in Tunisia initiated a knowledge and technology transfer project. The collaboration centered on introducing federated learning concepts, implementing distributed analytics through PADME as an operational PHT platform and exploring how Data Lakehouse architectures could support Tunisia’s emerging healthcare data landscape in the future.

This perspective article synthesizes lessons learned from this collaboration, discusses the opportunities and challenges encountered and describes how federated learning and Data Lakehouse infrastructures can be adapted for sensitive healthcare environments. We provide a critical and forward-looking viewpoint on how cross-regional partnerships can accelerate responsible digital healthcare innovation.

### 1.1 The Taz’iz partnership programme

The Arabic word “Ta’ziz” means “strengthening / consolidation” and has been chosen to clearly express the intended outcome of the Ta’ziz partnership programme. The programme provides spaces for dialogue on reform efforts at higher education institutions between Germany and the MENA region to promote joint teaching, research, administrative development and structured knowledge transfer as an important factor in social and political transformation.

Funding under the ‘Ta’ziz Short-Term Measures’ programme line is intended to promote the establishment and/or expansion of professional cooperation between German higher education institutions and higher education institutions from the MENA region. An additional aim is that existing partnerships and established scientific relationships that have already been funded are finalised or intensified/consolidated through specific and short-term measures.

Overall, the objectives of the Ta′ziz funding programme are:

1. Cooperation and transfer of knowledge between the participating higher education institutions and non-university partners in the areas of teaching, research and/or higher education management and/or transfer are initiated, intensified, expanded and/or consolidated.
2. Students, lecturers, (junior) scientists/ researchers and/or higher education management staff have acquired (inter)disciplinary and/or administrative competences.
3. The implementation of concepts and/or (knowledge-based) products relating to teaching, research and/or the reformative processes in the field of higher education management are initiated or intensified in ways that correspond to the local context and reflect the state of the art in science.

### 1.2 What the research collaboration has been about

In the scope of the Ta’ziz partnership, our project has been designed as a knowledge and technology transfer initiative to leverage from the experiences of the German research team in Cologne in building and deploying the Platform for Analytics and Distributed Machine Learning for Enterprises (PADME), a mature distributed analytics infrastructure that applies algorithms directly at data sources, enhancing data security and ensuring compliance with privacy regulations (1, 4). Developed in alignment with the Personal Health Train (PHT) approach, PADME enables the analysis of distributed data without transferring sensitive information, a key advantage for secure and privacy-preserving data collaboration.^2^

The aim of this knowledge transfer initiative is to facilitate the transfer of skills and bridge the gap between Tunisian academic research and practical applications in healthcare data by focusing on the implementation of federated learning and Data Lakehouse architectures as two advanced technologies with transformative potential in healthcare analytics.

**Figure 1:**
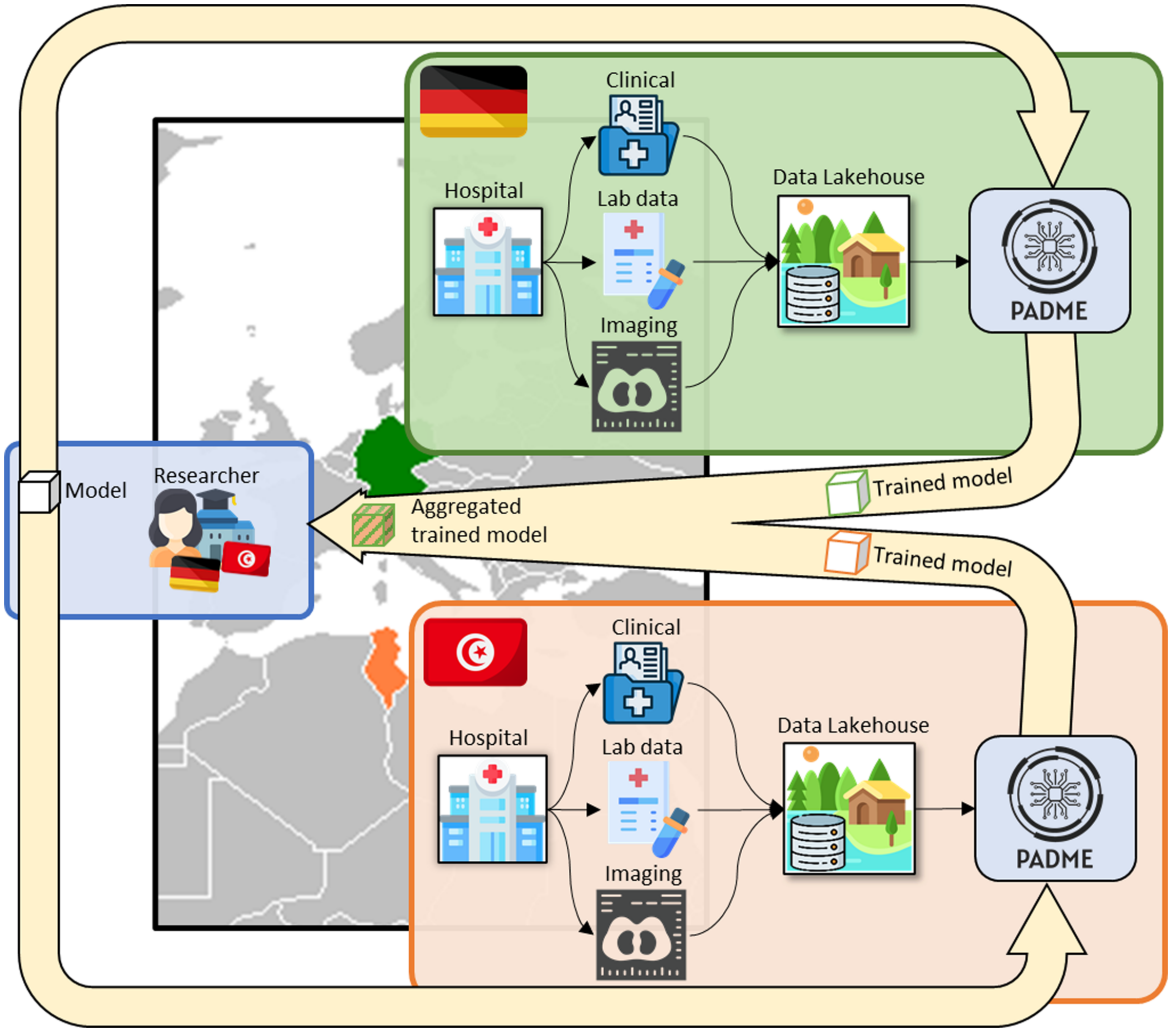
Integrating sensitive multi-modal hospital data from clinical routine through a Data Lakehouse for seamless data access for subsequent distributed analytics and federated learning approaches in PADME. Researchers define a model to train based on their research question, which is then distributed to the respective institutions for local model training on the patient data. Trained models are then returned and aggregated for an overall model trained in a privacy-preserving fashion on the distributed institutional data.

**Figure 2:**
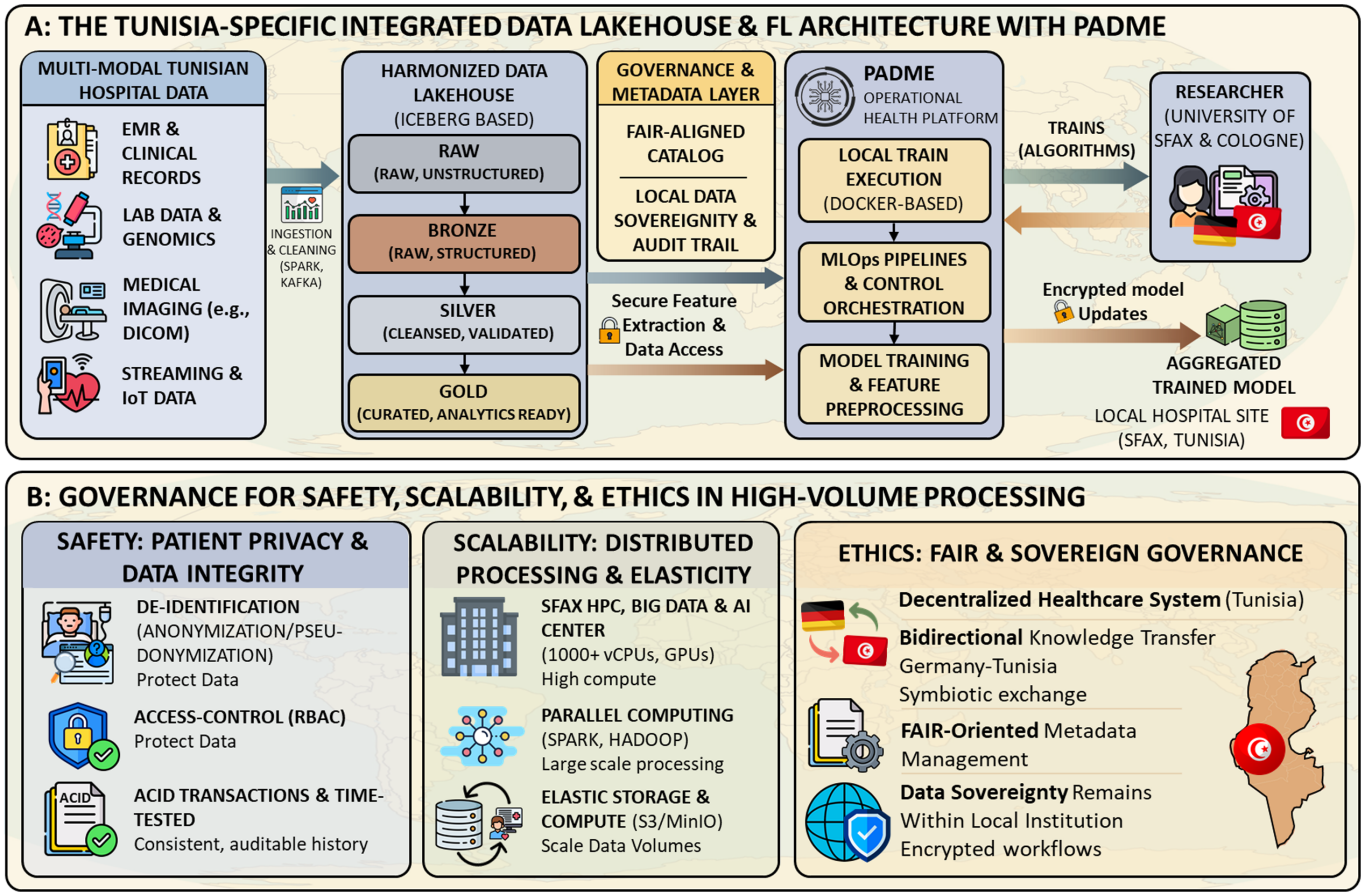
Integrated Data Lakehouse and PADME federated learning architecture for Tunisia: A workflow aligned with FAIR, scalable, and ethical standards.

While federated learning enables machine learning models to be trained across multiple data sources without transferring raw data, a Data Lakehouse combines the scalability of a data lake with the structured organization of a data warehouse, facilitating efficient data storage, access, and analysis for seamless federated learning approaches. In this context, multimodal healthcare data (clinical records, laboratory data, and imaging) are ingested and managed within the Lakehouse environment, while federated learning platforms such as PADME enable distributed model execution directly at each institution. This interaction establishes a structured data flow in which data remain locally stored and governed, while only model updates are exchanged and aggregated across participating institutions. The overall concept is visualized in (Figure 1). To further clarify the operational workflow of the proposed architecture—particularly the integration between the Data Lakehouse and federated learning components—an additional illustration is provided in Figure 2.

The project brings together the University Hospital of Cologne’s Biomedical Informatics Institute team and the Data Engineering and Semantics Research Unit at the University of Sfax. Each team brings distinct strengths: the German team contributes expertise in federated learning, distributed analytics, and data compliance based on research projects such as LeukoExpert (4) or the EU Horizon project BETTER (7).

The Data Engineering and Semantics Research Unit at the University of Sfax brings substantial expertise in Big Data system architecture, distributed computing, semantic data integration, and modern data management paradigms, forming a strong technical foundation for the development and management of Data Lakehouse infrastructures. Over the past decade, the team has designed and deployed several large-scale Big Data pipelines and mini-data-centre infrastructures, notably through national projects funded by the Tunisian Ministry of Higher Education and Scientific Research and multiple international collaborations, including two DAAD-funded initiatives.

A key strength of the Sfax team lies in its capacity to engineer end-to-end distributed data ecosystems, integrating ingestion, storage, distributed processing, governance, and semantic harmonization. In the SPEED-TUN national project on COVID-19 (2020–2021), the team conceived a complete Big Data architecture covering collection, ingestion, storage, analysis, and visualization of large-scale social data. This infrastructure relied on technologies such as Apache Kafka, Hadoop, Accumulo, Spark, Flume, Thrift, and Zookeeper, and incorporated ontology-driven semantic integration. The work builds on earlier research, notably the SNOWL ontology designed to unify heterogeneous social network data under a semantic RDF model (8), and illustrates the team’s operational capability to deploy distributed infrastructures, including a pilot mini-datacenter implemented within the Faculty of Sciences of Sfax.

The unit has also acquired extensive experience in managing heterogeneous, multimodal, and high-volume datasets, ranging from Big RDF ecosystems and IoT-generated streams to large-scale social network datasets. Key scientific contributions include advanced frameworks for semantic relatedness (9), ontology-driven semantic integration (10, 11), and knowledge-based analytics, which support FAIR-compliant metadata governance and schema harmonization—fundamental pillars of Data Lakehouse architectures.

In addition to semantic and Big Data integration, the team has contributed significantly to modern distributed data management paradigms. Their 2023 article, “Data Virtualization Enabling Distributed Data Architectures: Data Fabric and Data Mesh” (12), demonstrates how data virtualization, data fabric, and data mesh enable interoperable, domain-oriented, and decentralized data ecosystems—core architectural principles that underpin Lakehouse environments. This is complemented by the 2024 article “Data-driven journey: a data management paradigm-centric review and data mesh capabilities” (13), which offers a comprehensive analysis of data mesh capabilities and the evolution of distributed data governance models, further reinforcing the team’s expertise in scalable, decentralized data architectures suitable for health data environments.

The research unit has also produced advanced work in network representation learning, crucial for handling unstructured and graph-based data often stored within Lakehouse environments. This includes multi-view GNN-based recommendation systems (14), cross-social-network anchor-user modeling (15), and cross-platform data fusion methodologies (16), illustrating the team’s ability to handle highly heterogeneous, multimodal data through distributed analytical pipelines.

From an infrastructural perspective, the Sfax team has repeatedly implemented high-availability Big Data architectures using the Hadoop ecosystem, diverse NoSQL systems (HBase, Cassandra, Accumulo), distributed compute engines (Spark, Flink, Storm), and virtualization technologies such as Proxmox, Docker, and LXC/LXD, complemented by Infrastructure-as-Code tools like Ansible and Vagrant. Several engineering theses supervised by the team demonstrate hands-on capability in deploying distributed IaaS platforms, orchestrating fault-tolerant Hadoop clusters, and automating data pipelines—reflecting a deep practical understanding of Lakehouse-aligned principles such as scalable storage, distributed processing, metadata governance, schema evolution, and secure access.

The team’s technical competence has been reinforced through longstanding collaboration with German institutions via DAAD-funded projects. The 2016 project, “Transferring the Research Training Group experience in Social Media Research,” conducted with the University of Duisburg–Essen, introduced advanced German methodologies for distributed social media analytics and reproducible research workflows. The 2017 DAAD project, “Transferring the research networking and valuation experience as a bridge between academic and industrial issues,” conducted with German academic and industry-oriented partners, further strengthened the team’s capabilities in interoperable research networking, applied data management, and structured international collaboration. These partnerships laid a methodological and organizational foundation that directly supports current efforts in federated learning (PADME) and Data Lakehouse infrastructures.

Combined, these achievements demonstrate the Sfax team’s ability to design, deploy, and operate Lakehouse-ready infrastructures, manage complex multimodal healthcare data, and align with the FAIR principles. This positions the University of Sfax as a key regional partner for adapting and scaling Data Lakehouse and federated learning technologies across healthcare ecosystems in Tunisia and the broader MENA region.

The University of Sfax also benefits from the HPC, Big Data & AI Center, a high-performance computing facility installed and deployed by the Data Engineering and Semantics Research Unit. The center provides more than 1,000 vCPUs, large-capacity ECC RAM, NVIDIA GPUs, and high-speed storage and networking, offering a robust infrastructure for data-intensive workloads.^3^ This environment supports object storage, distributed compute clusters, and GPU-accelerated analytics—components that are essential for operating a modern Iceberg-based Data Lakehouse. By hosting the infrastructure locally, the team ensures strong data governance and sovereignty, allowing sensitive healthcare data to be processed securely while enabling large-scale preprocessing, multimodal analytics, and federated-learning experiments.

Through this collaboration, both teams aim to create a robust, privacy-compliant data infrastructure tailored for healthcare applications.

The collaboration within this project is structured into two major components: an internship program and a knowledge sharing workshop in Tunisia.

In the internship program, a group of Tunisian computer science students participated in a two-month internship involving a three-week long in-person visit in Germany, where they acquired hands-on training on developing MLOps pipelines and understanding privacy-preserving analytics and compliance workflows. In particular expertise was gained on configuring and deploying containerized PADME platform components, working with continuous Integration and Deployment (CI/CD) processes for federated learning environments and Data Lakehouse design principles and FAIR data management.

This practical training enabled students to work with realistic healthcare data pipelines for real-world applications of federated learning and Data Lakehouse architectures, equipping them with the technical skills and insights needed to address data challenges in healthcare.

After completing the internship, the students, in collaboration with German and Tunisian experts, organized a large-scale knowledge sharing workshop in Tunisia, engaging stakeholders from both academic and industrial ecosystems. This workshop focused on knowledge dissemination, targeting students and professionals across multiple disciplines, including data science, computer science, and healthcare informatics. The workshop included hands-on sessions, real-world healthcare use cases, and best practices in data governance and interoperability, fostering a comprehensive understanding of the project’s technological framework. The workshop played a pivotal role in disseminating knowledge beyond the project team, stimulating broader interest in federated analytics within the Tunisian ecosystem.

Overall, this project also focused on the transfer and acquisition of critical skills needed to manage a Data Lakehouse infrastructure within the context of healthcare data while adhering to the FAIR principles (Findable, Accessible, Interoperable, and Reusable). Furthermore, the initiative prioritized co-authoring scientific publications, enabling students and researchers from both countries to contribute to cutting-edge research in healthcare data management and analytics, disseminate findings to the global academic community, and strengthen their research profiles.

By focusing on capacity building, technical innovation, and knowledge dissemination, the project aimed to create a sustainable model for healthcare data management, fostering future collaboration between Germany and Tunisia.

## 2 Technical perspective: federated learning and Data Lakehouse approaches

Healthcare datasets are highly sensitive and cannot easily be centralized due to privacy regulations, institutional policies, and ethical constraints. Federated learning addresses these challenges by ensuring that patient data never leave institutional boundaries and conducted analytics are always embedded within local IT governance structures, for instance at respective hospitals. Doing so enables multi-site analyses without compromising data sovereignty and supports model training even when individual datasets of institutions are small, heterogenous or siloed.

For regions such as MENA with countries such as Tunisia with evolving national privacy regulations (17), and varying infrastructures across institutions, federated learning offers a pragmatic and scalable approach to collaborative analytics. In Tunisia, this is particularly relevant due to the decentralized healthcare system, heterogeneous hospital information systems, and fragmented data ecosystems, which require architectures that preserve institutional autonomy while enabling coordinated analytics at national scale (18).

### 2.1 The personal health train: bringing algorithms to the data

The Personal Health Train (PHT) paradigm operationalizes the core idea of distributed analytics and federated learning: instead of moving data to the algorithm, the PHT paradigm shift brings the algorithms (“Trains”) to the data (“Stations”) as a solution for data privacy-related challenges. The PHT concept is designed to protect the patient’s sensitive data by ensuring data providers retain full control over their data and that tasks are always executed in isolated, auditable environments. Moreover, researchers are provided with only essential patient medical data needed for their research and strict access controls ensure that only authorized individuals can access the sensitive patient data. The PHT provides tools for researchers to analyze gathered data and generate insights without compromising the privacy of the individuals whose data is being used.

### 2.2 PADME: an operational implementation of the PHT

The Platform for Analytics and Distributed Machine Learning for Enterprises (PADME) is a mature, production-ready implementation of the PHT concept. PADME implements various security measures, such as encryption, access control, and auditing, to ensure the confidentiality, integrity, and availability of patient data. In combination with the Data Lakehouse architecture, these mechanisms support safe and ethical data processing by maintaining strict data locality, enforcing controlled access and auditability, and aligning with FAIR data governance principles. Furthermore, the distributed design of both the Lakehouse and federated learning workflows enables scalability by supporting increasing data volumes and heterogeneous data sources across institutions.

The PADME design targets real-world healthcare settings by integrating Docker-based container execution to facilitate computations on heterogeneous hardware configuration or systems. Moreover, encrypted Train packaging and execution increases security and a central Station registry leads the onboarding and managing of institutions for control and auditability. On a technical level, secrets are managed on a vault-basis and metadata is monitored via Telegraf and a global metadata store. The Central service coordinates the train orchestration, access control and logging.

PADME Stations operate autonomously by periodically polling the Central Station for approved Train requests, pulling and decrypting Trains, executing Trains locally, and subsequently encrypting and pushing results back to the Central Station. Trains are designed by researchers for their respective distributed analytics or federated learning research questions and are approved by the infrastructure provider. Institutional sovereignty is preserved through the approval process of Trains and the one-way communication design, which ensures that the Central Service cannot initiate direct activity on Stations.

### 2.3 Data Lakehouse architectures and FAIR principles

While federated learning focuses on secure computation across distributed datasets, Data Lakehouse architectures address the equally critical need for scalable, governed, and interoperable data storage. In healthcare environments—where data originates from electronic health records, laboratory systems, imaging devices, IoT sources, and administrative workflows—traditional architectures such as relational warehouses or monolithic Hadoop clusters struggle to accommodate the heterogeneity, volume, and governance requirements of modern clinical analytics. Data Lakehouses fill this gap by unifying the flexibility of data lakes with the transactional guarantees and metadata richness of data warehouses, thereby enabling reliable, long-term management of multimodal clinical datasets.

Within this collaboration, the Tunisian team is developing a modern Data Lakehouse architecture centered on Apache Iceberg, a state-of-the-art open table format designed for large-scale analytical workloads. Apache Iceberg brings essential capabilities for data within sensitive domains such as healthcare, including ACID transactions, time-travel for historical reproducibility, schema evolution without downtime, and hidden partitioning that automatically optimizes query performance. These features ensure that evolving patient records, changing clinical codification schemes, or new data modalities can be incorporated seamlessly without breaking existing pipelines or analytic models.

The Iceberg-based Lakehouse is composed of several complementary technologies. At the processing layer, Apache Spark serves as the primary distributed engine for ingestion, cleaning, transformation, and feature engineering. Its native integration with Iceberg allows efficient reading and writing of very large datasets, enabling reproducible pipelines for preparing machine-learning-ready feature stores. At the storage layer, Iceberg tables are hosted on scalable object storage systems such as MinIO or any S3-compatible backend, ensuring decoupled compute and storage while maintaining high availability and elasticity.

The governance and metadata layer relies on Iceberg catalogs, such as Hive Metastore, Nessie, or Glue-compatible catalogs, which maintain consistent table definitions, schema history, snapshots, and audit logs. This metadata layer reinforces the FAIR principles by making data assets Findable through catalog indexing, Accessible through standard SQL interfaces, Interoperable via open table formats and structured metadata, and Reusable thanks to high-quality documentation, schema versioning, and lineage capabilities. Workflow orchestration tools such as Apache Airflow or Dagster automate ingestion, transformation, and validation tasks, ensuring that the Lakehouse operates reliably and reproducibly. When real-time ingestion is required, technologies such as Apache Kafka support low-latency streaming pipelines from hospital systems or digital health platforms.

The adoption of a Data Lakehouse architecture brings multiple benefits to emerging healthcare data ecosystems. First, it enables harmonization of highly heterogeneous multimodal datasets—including tabular clinical records, imaging metadata, streaming device data, and genomic information—within a single governed environment. Second, Iceberg’s strong governance and reproducibility features allow healthcare institutions to maintain auditable histories of data modifications, an essential capability in contexts involving patient privacy, ethics, and regulatory compliance. Third, because lakehouses expose a unified analytical interface, they significantly simplify downstream processing for advanced analytics, including preparing federated learning features or aggregating institution-specific data without breaking sovereignty constraints. Finally, the decoupling of compute and storage allows the system to scale organically and cost-effectively, making the architecture sustainable for the long term in resource-limited health systems. In addition to scalability and sustainability considerations, cost remains a critical factor for real-world deployment. From a cost perspective, implementing a Data Lakehouse combined with federated learning requires investments in storage, compute infrastructure, networking, and human capacity building. However, compared to centralized architectures, federated approaches reduce the need for large-scale data transfer and centralized storage, leading to more efficient cost scaling as data volume increases. This makes the proposed architecture particularly suitable for resource-constrained environments such as Tunisia.

In combination, these technological components and architectural principles position the Iceberg-based Lakehouse as a powerful foundation for modern hospital data platforms in Tunisia. The approach enables both robust governance and flexible innovation, ensuring that multimodal patient data can be securely harmonized, analyzed, and leveraged for next-generation distributed analytics, including federated learning with PADME.

## 3 Discussion

### 3.1 Challenges and the potential for uptake and adoption in Sfax

The collaboration revealed several promising factors for coupling Data Lakehouses and federated learning for further adoption in Cologne in Germany and the merging ecosystems in Sfax and Tunisia as a whole.

Many hospitals in Tunisia rely on manual data transfer, commercial consumer cloud storage platforms such as Google Drive for institutional data sharing due to a lack of data management expertise, further posing risks to patients’ privacy and data integrity due to a lack of governance capacities (19). These practices pose not only major risks to data privacy and integrity, but are also unsustainable as data volumes grow, for instance due to accumulated patient data in the form of large genomics or imaging files. Especially in the increasingly more decentralized healthcare system in Tunisia as an effort to decrease regional health inequalities, a lack of standardization and fragmentation of patient data across hospital systems poses further obstacles (18).

Beyond the challenges described above, the situation in Sfax reveals a number of structural and technical factors that directly influence the adoption of Data Lakehouse architectures and federated learning technologies. Despite the strong research capacity at the University of Sfax, many healthcare institutions in the region continue to operate with limited IT infrastructures, often lacking high-availability servers, redundant storage, secure internal networks, or virtualization environments. Hospital information systems are frequently outdated, fragmented, or deployed in isolation, which restricts interoperability and complicates the implementation of modern data-intensive workflows. These infrastructural limitations make it difficult for hospitals to ingest, store, and manage large multimodal patient datasets—particularly imaging, genomics, and real-time sensor streams—which are becoming increasingly central to precision medicine and digital health.

A second major challenge concerns data governance maturity. Most hospitals and regional health authorities in Tunisia do not yet have established workflows for metadata documentation, versioning, lineage tracking, or structured access control. This situation also reflects the absence of comprehensive regulatory and legal frameworks governing health data management and sharing. Strengthening national legislation aligned with international standards for data protection and interoperability would provide a critical foundation for enabling secure, privacy-preserving analytics and cross-institutional collaboration in Tunisia and the broader MENA region. These challenges can be structured into key gaps, including: (i) infrastructure limitations related to scalable storage, compute, and networking; (ii) governance gaps such as lack of metadata standards, lineage tracking, and access control; (iii) data fragmentation and scarcity across decentralized systems; and (iv) limited human capacity in data engineering and governance.

Data flows are often informal, undocumented, and dependent on individual staff members, resulting in inconsistent quality and limited reproducibility. This lack of governance is precisely why the current collaboration sought to demonstrate the value of the medical data integration center (MeDIC) infrastructure in Cologne^4^—as a model that integrates workflow automation, semantic data harmonization, secure access layers, and federated learning capabilities. The experience highlighted the importance of institutionalizing similar governance practices in Sfax to ensure FAIR principles, privacy-preserving analytics, and compliance with emerging national and regional regulations.

A third challenge relates to human resources and organizational culture. Many hospitals IT teams in Sfax have limited exposure to distributed systems, containerization, or modern data engineering tools such as Spark, Iceberg, or Kubernetes. The absence of dedicated data stewards, MLOps engineers, or governance officers further complicates the adoption of advanced analytical infrastructures. While the collaboration helped build foundational capacities, sustained training and long-term institutional support will be essential to operationalize a Lakehouse-enabled healthcare ecosystem.

Finally, large-scale adoption will require long-term, government-driven investment. Implementing secure Data Lakehouses across hospitals, combined with federated learning nodes, cannot rely solely on short-term research funding. Institutions will need stable financial support to secure hardware, maintain cloud or on-premise resources, upgrade networks, and invest in continuous capacity building.

Beyond financial investment, these findings highlight the need for coordinated national strategies in Tunisia and across MENA countries to modernize healthcare data ecosystems. In particular, governments should promote interoperability standards, structured data governance frameworks, and secure data-sharing mechanisms adapted to decentralized healthcare systems. Highlighting these needs is strategically important not only for ensuring sustainability of the current DAAD-funded project but also for motivating follow-up initiatives—both bilateral and national—that could scale the model across the Tunisian healthcare system.

Overall, the experience in Sfax shows that while the scientific and technical foundations for federated learning and lakehouses are strong, the regional ecosystem requires coordinated efforts in IT infrastructure modernization, governance standardization, capacity building, and long-term investment to fully realize their potential. Future improvements should focus on strengthening governance frameworks, standardizing data models, improving interoperability, investing in scalable infrastructure, and developing human capacity through targeted training and institutional support.

### 3.2 Lessons learnt and the future plan for collaboration

The collaboration between the University of Cologne and the University of Sfax generated several important lessons that will guide future joint activities and the long-term development of distributed data infrastructures in Tunisia. A key insight emerging from the project is the critical importance of hands-on training. Practical exposure to technologies such as Apache Iceberg, Spark, metadata catalogs, and federated learning orchestration provided students and researchers with a level of understanding that cannot be achieved through theoretical instruction alone. Experimenting directly with the PADME framework, implementing ingestion and processing pipelines, and deploying Lakehouse components revealed technical nuances—particularly regarding storage formats, schema evolution, and system interoperability—that participants could only appreciate through real-world exercises.

A second lesson relates to the value of proof-of-concept (PoC) implementations. A small-scale prototype served as an essential mechanism for identifying design gaps, uncovering integration friction points, and validating feasibility. Connecting PADME with an Iceberg-based Lakehouse architecture exposed several technical challenges: input preprocessing workflows needed standardization, model execution environments required tailored containerization, and metadata catalogs had to be made accessible in a consistent manner across systems. These insights will directly inform the next iteration of the architecture and highlight the need for harmonized interfaces between federated learning platforms and Lakehouse storage layers.

The collaboration also underscored the importance of involving administrative and IT departments from the outset. Many of the obstacles encountered—such as network restrictions, server access policies, and approval processes for deploying new software—are institutional rather than purely technical. While such governance structures are common in hospital environments, the project demonstrated that early engagement with IT services, legal teams, and administrative decision-makers is crucial for ensuring sustainability, regulatory alignment, and smooth deployment. This experience was particularly relevant for the Tunisian context, where IT teams often operate under resource constraints and where administrative approval cycles can significantly delay innovation unless proactively managed.

Another key lesson is the benefit of bidirectional knowledge exchange. The collaboration was not a one-way transfer of expertise from Cologne to Sfax and instead, both sides contributed complementary strengths. The German team provided insights into workflow automation, secure federated analytics, and the operational experience of MeDIC. The Tunisian team brought deep expertise in Big Data architecture, virtualization, semantic integration, and system deployment under resource-constrained environments. This reciprocal exchange enriched the project outcomes and created a shared understanding that will continue to underpin future work.

Finally, the project highlighted the central role of interoperability, both in terms of data harmonization and system integration. Ensuring consistent schemas, stable metadata definitions, and compatible preprocessing workflows is essential for federated learning. Likewise, integrating PADME with a Lakehouse environment requires alignment at the API level, data format level, and orchestration level. The challenges encountered by the students—ranging from container incompatibilities to schema mismatch issues—demonstrated the need for common standards, shared workflows, and robust middleware to ensure seamless communication between components. This will be a primary focus of future developments.

Looking ahead, both institutions plan to strengthen and expand the collaboration through follow-up research projects, joint supervision of advanced students, co-development of interoperable PoC architectures, and the establishment of training programs focused on Lakehouses, federated learning, and healthcare data governance. A long-term objective is to create a replicable and scalable distributed analytics framework that can support real-world hospital use cases across Tunisia, while aligning with international best practices and benefiting from ongoing German-Tunisian academic cooperation. This includes a progressive deployment strategy, starting with pilot implementations, followed by institutional scaling and broader integration within the Tunisian healthcare system, taking into account technical, organizational, and regulatory constraints.

### 3.3 Future directions for federated learning in Tunisia and the MENA region

The work achieved in this project lays the groundwork for future developments towards a more connected healthcare infrastructure in the highly decentralized healthcare system in Tunisia. Beyond its academic and technical contributions, this work provides important insights for policymakers in Tunisia and across the MENA region. The findings highlight the need for structural reforms in healthcare data management, including the establishment of interoperable digital infrastructures, the adoption of standardized data governance frameworks, and the development of legislation enabling secure and privacy-preserving data sharing across institutions.

A subsequent established distributed analytics and federated learning ecosystem in the future could enable broad access to otherwise highly sensitive patient data not only to academic institutions, but also other stakeholders such as small and medium sized enterprises (SME), for example to evaluate products such as developed clinical decision support systems with real patient data in the process of the clinical evaluation (20). Such policy-driven transformation is essential to fully leverage federated learning and Data Lakehouse architectures as enablers of resilient, data-driven healthcare systems in decentralized contexts across the MENA region.

Tunisia, as an associated country to the European Union’s Horizon Europe program, can use the opportunity to engage Tunisian scientists and researchers to participate in the program under the same conditions as those from EU member states, making it eligible for the ‘Widening’ funding opportunities, amongst which count Teaming and Twinning initiatives. While for the former the aim is to help create new, high-quality centres of excellence in a widening country, as Tunisia in our case, by partnering with a leading institution, the latter focus on strengthening specific research fields through knowledge transfer and networking. Both form part of our future collaboration plans and joint research agendas and, in this respect, our bilateral project that has been funded by DAAD has been vital for sustaining continuity as it allowed both teams to build trust, share knowledge, and compound incremental findings into what we aspire to be long-term, transformative research impacts and sustainable institutional relationships.

## Data Availability

The original contributions presented in the study are included in the article/supplementary material, further inquiries can be directed to the corresponding author.

## Footnotes

1 https://www.daad.de/en/information-services-for-higher-education-institutions/further-information-on-daad-programmes/taziz-partnership/ (visited on November 28th, 2025).

2
https://padme-analytics.de/

3
https://hpc.univ-sfax.tn

4 The University Hospital of Cologne and the Faculty of Medicine of the University of Cologne are involved in the Medical Informatics Initiative (MI-I) and the Network of University Medicine (NUM), which are funded by the Federal Ministry of Education and Research. The MI-I and NUM initiatives aim to leverage the opportunities of digitalization in medicine for both healthcare provision and research. To this end, Medical Data Integration Centers (MeDICs) have been established and networked at the participating university hospitals and partner institutions. Over the past few years, these centers have created the necessary conditions to make research and healthcare data available across different locations.
